# Multiple Firing Patterns in Coupled Hindmarsh-Rose Neurons with a Nonsmooth Memristor

**DOI:** 10.1155/2020/8826369

**Published:** 2020-11-07

**Authors:** Xuerong Shi, Zuolei Wang

**Affiliations:** School of Mathematics and Statistics, Yancheng Teachers University, Yancheng 224002, China

## Abstract

A model is introduced by coupling two three-dimensional Hindmarsh-Rose models with the help of a nonsmooth memristor. The firing patterns dependent on the external forcing current are explored, which undergo a process from adding-period to chaos. The stability of equilibrium points of the considered model is investigated via qualitative analysis, from which it can be gained that the model has diversity in the number and stability of equilibrium points for different coupling coefficients. The coexistence of multiple firing patterns relative to initial values is revealed, which means that the referred model can appear various firing patterns with the change of the initial value. Multiple firing patterns of the addressed neuron model induced by different scales are uncovered, which suggests that the discussed model has a multiscale effect for the nonzero initial value.

## 1. Introduction

As the building elements of the nervous system, the neuron is the most fundamental unit in neural processing. Research on the nonlinear dynamics of neurons is crucial for revealing the mechanisms underlying perception and transmission of neural information.

In 1952, to describe the ionic conductance dynamics of the giant axon, Hodgkin and Huxley established the Hodgkin-Huxley (HH) neuron model [1], which started the research on the neuron model. From then on, other simplified neuron models were proposed successively, which mainly explained lots of ion channels, various synapses, and spatial geometry of individual cells, such as the FitzHugh-Nagumo (FHN) model [2] depicting a prototype of a neuron, the Hindmarsh-Rose (HR) model [3] simulating the characteristics of neurons in the hippocampus of the brain, the Morris-Lecar (ML) model [4] obtained in the research on muscle fiber of Arctic goose, the Chay model [5] as a new theoretical model with unity based on many different types of excitable cells, and the Izhikevich neuron model [6] regarded as a mathematical simplification of the HH model using a binary tree. These models represented a variety of neurons. These neuron models have demonstrated different electrical activities and attracted many researchers' attention. For example, the FHN neuron model exhibits discontinuous transition between different oscillations [7] and double coherence resonance induced by phase noise [8]; the HH neuron model displays evoking spiking caused by enough noise intensity [9], chaotic resonance dependent on current intensity [10], and extrinsic stochastic resonance caused by ion shot noise [11]; in the presence of periodic input, the HR neuron model can show nonlinear resonance behavior [12], periodic and chaotic firing patterns [13], transition between chaotic firing and periodic firing [14], and bursting phenomenon [15]; the Izhikevich neuron model can appear chaotic resonance [16, 17]; the ML neuron model can exhibit mono- and bistable dynamic regimes [18] and responses to two temperature-sensitive ion channels, calcium and leak current, respectively [19]. These classical models and their dynamical analysis are motivating researchers to develop more realistic or refined neuron models.

In recent years, improving the classical neuron models by different schemes, such as by introducing the electrical magnetic effect, has received intense attention. For example, considering magnetic flux and Gaussian white noise, a modified Izhikevich model was set up and diverse firing patterns for a different set of parameters were brought to light [20]. An improved HH neuron model was raised, and the firing frequency dependent on the external forcing current was analyzed [21]. A memristor-based HH neuron model was constructed, and multiple patterns of electrical activities as well as stochastic resonance were detected [22]. A stochastic HH model was brought up, and the effects of ionic channel blockage on the electrical activities of it were studied [23]. Kinds of improved FHN models [24–26] came up, and the complicated dynamics were revealed, such as noise-enhanced stability and resonance [24], hidden extreme multistability [25], and subcritical Hopf bifurcation and stochastic resonance [26]. The ML neuron model was improved [27–29] along with the dynamical properties being explored, like the memristor synapse-based ML model with abundant periodic and chaotic bursting [27], the random dynamical behavior driven by Gaussian white noise [28], and the stochastic hybrid ML model with the extended parameter regime of oscillations related to noise [29]. In particular, many researchers paid much attention to the well-known HR model and revised it utilizing various methods [30–38], including impulsive effect and period-adding bifurcation in a hybrid HR model [30]; alternating current-induced coexisting behaviors of asymmetric busters in an external alternating current injected HR model [31]; diversity of firing patterns in a memristor-coupled HR model [32]; multiple firing patterns dependent on the complex electrophysiological condition found in a memristor HR model [33]; coherence resonance induced by phase noise in a 3D memristor-based HR model [34]; the relationship between the energy and the firing pattern in a type of memristor-based HR model [35]; the coexisting phenomenon of diverse firing patterns in a 5D memristor-coupled HR neuron model [36]; hyperchaotic behavior in a kind of neuron model with discontinuous magnetic induction [37]; and different types of firing patterns in a modified Hindmarsh-Rose model, such as square-wave bursting, chattering, fast spiking, periodic spiking, and mixed-mode oscillations [38]. Additionally, a novel neuron model, the Wang-Zhang model, built directly at the neuron level was brought forth [39]. Research results [40, 41] suggested that the Wang-Zhang model is equivalent to the HH neuron model from the perspective of neural energy calculation and energy coding. The aforementioned results indicate that to better show electrical activity of the neuron system, many improved neurons were brought up from different perspectives and various dynamical phenomena were gained. It is worth mentioning that among the referred methods to improve the neuron model, memristor coupling was an important approach when considering the effect of the electric field. However, the memristor coupling was mainly about a smooth memristor. Nonsmooth memristor-coupled neurons as well as the dynamics were hardly reported.

Actually, there is often a nonsmooth memristor, which can be used and make the system appear complicated dynamics. Inspired by this idea, in this paper, a nonsmooth memristor is in consideration in the HR neuron model and firing patterns of it are to be discovered. Other parts of this paper are arranged as follows. In Section 2, a novel neuron model is described by coupling two HR neurons using a nonsmooth memristor and different firing patterns are given when changing the external forcing current. In Section 3, the equilibrium points along with their stabilities of the introduced model are analyzed quantitatively. In Section 4, the coexistence of different firing patterns dependent on the initial values is exhibited via numerical simulations. In Section 5, the multiscale feature of the neuron model is discussed. Some conclusions are drawn in Section 6.

## 2. Nonsmooth Memristor-Coupled Neuron Model

### 2.1. Model Descriptions

In this section, a nonsmooth memductance function [42] is considered to be

(1) $$\begin{array}{c} W\left(\varphi \right)=\alpha +3\beta \left|\varphi \right|, \end{array}$$

which is an ideal and active flux-controlled memristor with absolute value nonlinearity, where *α* and *β* are two memristor parameters with positive values.

When there exists a membrane potential difference between two neurons, electromagnetic induction current will be sensed. For this reason, memristor synapse can be applied to characterizing the dynamics caused by the membrane potential difference. Consequently, on the basis of the 3D HR model [43], a model involving two neurons coupled with a nonsmooth memristor can be presented as

(2) $$\begin{array}{c} \left\{\begin{array}{c} \dot{x}=y-{ax}^{3}+{bx}^{2}-z+I_{\mathrm{ext}}+kW\left(\varphi \right)\left(x-u\right),\\ \dot{y}=c-{dx}^{2}-y,\\ \dot{z}=r\left[S\left(x+1.6\right)-z\right],\\ \dot{u}=v-{au}^{3}+{bu}^{2}-w+I_{\mathrm{ext}}+kW\left(\varphi \right)\left(x-u\right),\\ \dot{v}=c-{du}^{2}-v,\\ \dot{w}=r\left[S\left(u+1.6\right)-w\right],\\ \dot{\varphi }=\left(x-u\right)-\varphi , \end{array}\right. \end{array}$$

where *x* and *u* represent the membrane potentials in coupled neurons, *y* and *v* mean the exchanges of ions in coupled neuron membranes, and *z* and *w* denote the adaptation currents. *I*_ext_ is the external forcing current. *k* is the coupling strength. *x* − *u* expresses the difference of membrane potential. System parameters *a* and *b* are the fitting coefficients of a cubic function which is used to describe the rate of change of membrane potential. The values of *c* and *d* are required to ensure the time course of adaptation current in voltage-clamp condition. *r* and *S* mean the corresponding parameters relative to a short depolarizing current and are utilized to depict the change of adaptation current.

### 2.2. Firing Patterns Affected by the External Forcing Current

**Figure 1:**
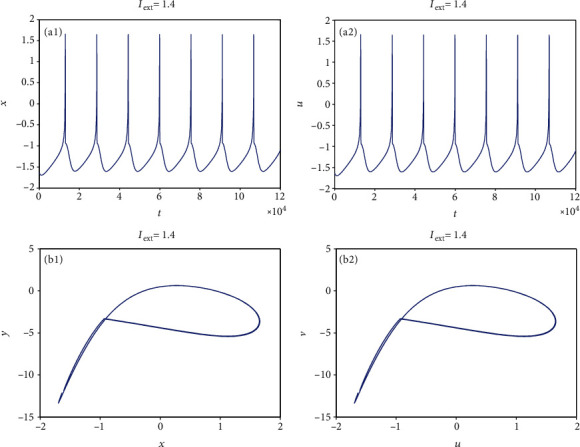
Period-1 firing pattern of model (2) when *I*_ext_ = 1.4 and *k* = 0.1. (a1 and a2) Firing patterns of membrane potentials. (b1 and b2) Phase portraits.

**Figure 2:**
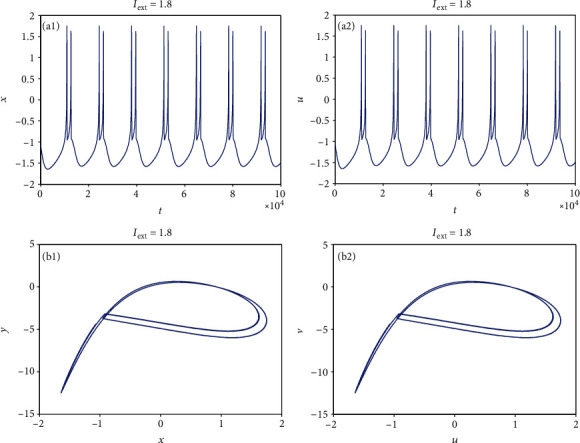
Period-2 firing pattern of model (2) when *I*_ext_ = 1.8 and *k* = 0.1. (a1 and a2) Firing patterns of membrane potentials. (b1 and b2) Phase portraits.

**Figure 3:**
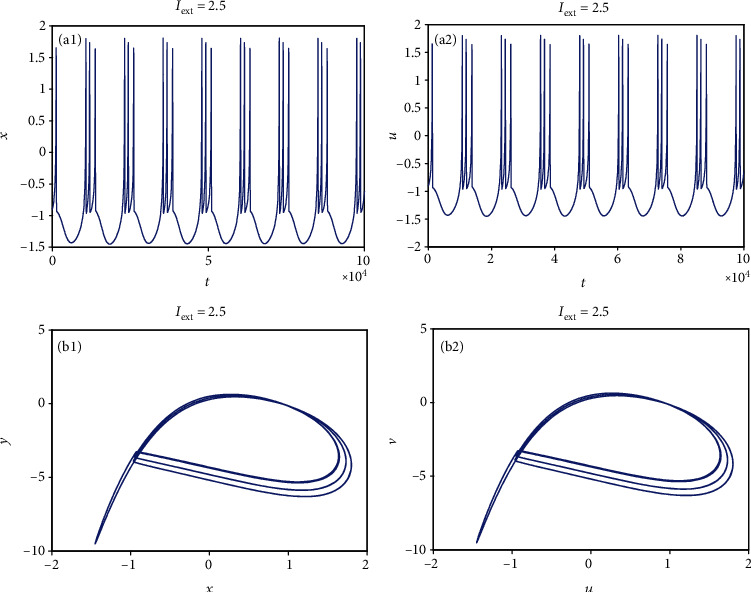
Period-3 firing pattern of model (2) when *I*_ext_ = 2.5 and *k* = 0.1. (a1 and a2) Firing patterns of membrane potentials. (b1 and b2) Phase portraits.

**Figure 4:**
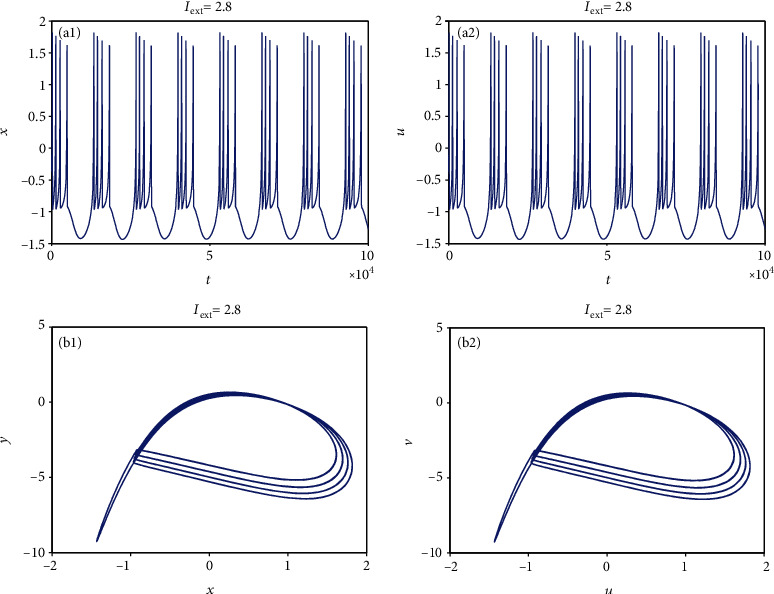
Period-4 firing pattern of model (2) when *I*_ext_ = 2.8 and *k* = 0.1. (a1 and a2) Firing patterns of membrane potentials. (b1 and b2) Phase portraits.

**Figure 5:**
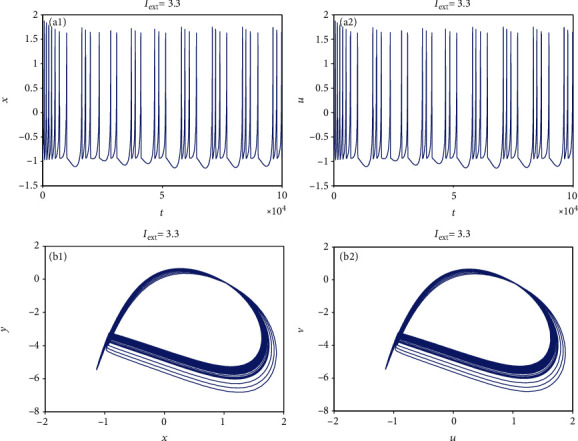
Chaotic firing pattern of model (2) when *I*_ext_ = 3.3 and *k* = 0.1. (a1 and a2) Firing patterns of membrane potentials. (b1 and b2) Phase portraits.

**Figure 6:**
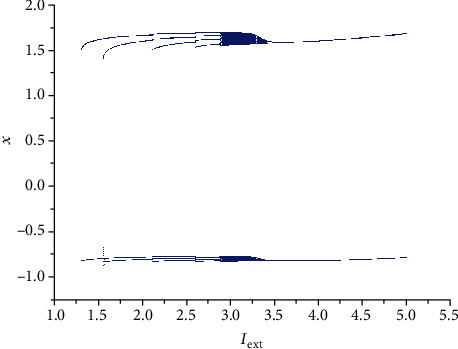
Bifurcation of membrane potential *x* (Poincare section at *y* = −2.5) in model (2) with *k* = 0.1 and bifurcation parameter *I*_ext_.

From Section 2.1, it can be known that by adjusting the rate of change of membrane potential, voltage-clamp condition, and change of adaptation current to an appropriate state, the system parameters can be chosen as *a* = 1.0, *b* = 3.0, *c* = 1.0, *d* = 5.0, *r* = 0.006, and *S* = 4.0. With these parameter values, the 3D HR model [43] can demonstrate complicated dynamics. To explore the firing patterns of model (2), the coupling strength is taken as *k* = 0.1 and the initial value is considered *x*_0_ = (0, 0, 0, 0, 0, 0, 0), and then the sampled time series of neuron model (2) are calculated and drawn in Figures 1–5, from which it can be seen that the addressed model (2) appears multiple firing patterns with the change of the external forcing current *I*_ext_ while other parameters are kept unchanged. Specifically, for *I*_ext_ = 1.4, 1.8, 2.5, 2.8, neuron model (2) displays firing patterns of period-1, period-2, period-3, and period-4, respectively. Namely, it shows an adding-period phenomenon. But for *I*_ext_ = 3.3, neuron model (2) comes into a chaotic state. To better characterize this process of change, Figure 6 draws the bifurcation of membrane potential *x* with bifurcation parameter *I*_ext_, which confirms the results of Figures 1–5. As has been noted, the firing patterns of model (2) are similar to the characteristics of the HR neuron model coupled with a smooth memristor [44].

## 3. Equilibrium Points and Their Stability Analysis

In this section, system parameters of neuron model (2) are also chosen as *a* = 1.0, *b* = 3.0, *c* = 1.0, *d* = 5.0, *r* = 0.006, *S* = 4.0, *α* = 4.0, *β* = 5.0, and *I*_ext_ = 3.0, with which model (2) is provided with a chaotic feature. The equilibrium points of (2) can be determined as

(3) $$\begin{array}{c} A=\left(\delta_{1},c-d\delta_{1}^{2},S\left(\delta_{1}+1.6\right),\delta_{2},c-d\delta_{2}^{2},S\left(\delta_{1}+1.6\right),\delta_{1}-\delta_{2}\right), \end{array}$$

where *δ*_1_ and *δ*_2_ can be regarded as the intersection points of function curves

(4) $$\begin{array}{c} F_{1}\left(\delta_{1},\delta_{2}\right)=\delta_{1}^{3}+2\delta_{1}^{2}+4\delta_{1}+5.4-I_{\mathrm{ext}}-k\left(\delta_{1}-\delta_{2}\right)\left(\alpha +3\beta \left|\delta_{1}-\delta_{2}\right|\right)=0, \end{array}$$

(5) $$\begin{array}{c} F_{2}\left(\delta_{1},\delta_{2}\right)=\delta_{2}^{3}+2\delta_{2}^{2}+4\delta_{2}+5.4-I_{\mathrm{ext}}+k\left(\delta_{1}-\delta_{2}\right)\left(\alpha +3\beta \left|\delta_{1}-\delta_{2}\right|\right)=0. \end{array}$$

**Figure 7:**
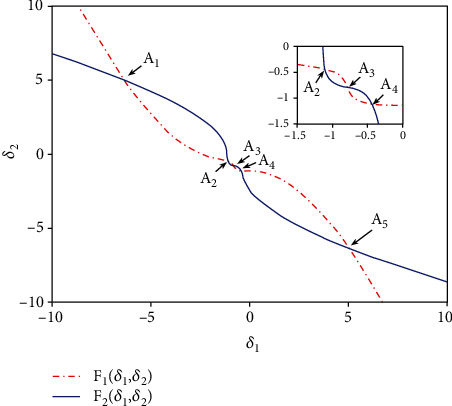
Function curves of (4) and (5) as well as the intersection points when *k* = 0.1. The red curve corresponds to function (4), and the blue one is for function (5). *A*_1_, *A*_2_, *A*_3_, *A*_4_, and *A*_5_ are five intersection points.

**Figure 8:**
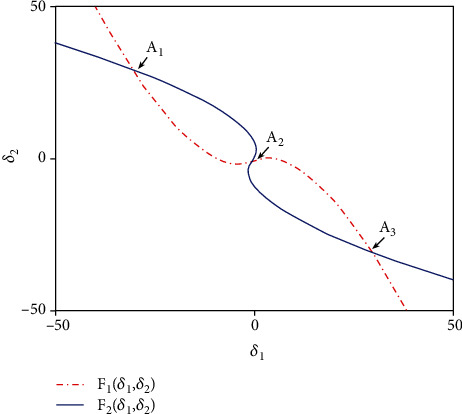
Function curves of (4) and (5) as well as the intersection points when *k* = 0.5. The red curve corresponds to function (4), and the blue one is for function (5). *A*_1_, *A*_2_, and *A*_3_ are three intersection points.

It can be solved via the graphic analytic method, which is a method to solve the problem in geometry. According to the conditions and conclusions of the problem to be solved, one or more basic graphics of it are found by analyzing. Then, the properties of these basic graphics are applied to solve the problem. Because (*δ*_1_, *δ*_2_) is believed as the intersection point of function curves (4) and (5), it can be obtained by picturing the curves of (4) and (5). Therefore, the intersection coordinate (*δ*_1_, *δ*_2_) can be achieved. Substitute the values of *δ*_1_ and *δ*_2_ into (3), and then the nonzero equilibrium points of model (2) can be gained. To this end, we take *k* = 0.1 and *k* = 0.5 as two examples. The intersection points of (4) and (5) can be calculated numerically and pictured in Figures 7 and 8, respectively, which indicate that for *k* = 0.1 and *k* = 0.5, the number of intersection points of (4) and (5) is 5 and 3, respectively. It means that the value of coupling coefficients has an effect on the number of intersection points. That is to say, the number of nonzero equilibrium points of model (2) is relative to the value of the coupling coefficient *k*.

To judge the stabilities of the equilibrium points, the Jacobian matrix of model (2) at *A* is yielded as

(6) $$\begin{array}{c} P=\left[\begin{array}{ccccccc} h_{1} & 1 & -1 & h_{2} & 0 & 0 & h_{3}\\ -2dx & -1 & 0 & 0 & 0 & 0 & 0\\ rS & 0 & -r & 0 & 0 & 0 & 0\\ h_{2} & 0 & 0 & h_{4} & 1 & -1 & -h_{3}\\ 0 & 0 & 0 & -2du & -1 & 0 & 0\\ 0 & 0 & 0 & rS & 0 & -r & 0\\ 1 & 0 & 0 & -1 & 0 & 0 & -1 \end{array}\right], \end{array}$$

where

(7) $$\begin{array}{c} h_{1}=-3a\delta_{1}^{2}+2b\delta_{1}+k\left(\alpha +3\beta \left|\delta_{1}-\delta_{2}\right|\right),\\ h_{2}=-k\left(\alpha +3\beta \left|\delta_{1}-\delta_{2}\right|\right),\\ h_{3}=3k\beta \left(\delta_{1}-\delta_{2}\right)\mathrm{sgn}\left(\delta_{1}-\delta_{2}\right),\\ h_{4}=-3a\delta_{2}^{2}+2b\delta_{2}+k\left(\alpha +3\beta \left|\delta_{1}-\delta_{2}\right|\right). \end{array}$$

For the given coupling strengths *k* = 0.1 and 0.5, the values (*δ*_1_, *δ*_2_) of the equilibrium points are calculated and listed in Tables 1 and 2. Meanwhile, utilizing the Jacobian matrix (6), the corresponding eigenvalues at *A* are computed and also given in Tables 1 and 2. By doing so, the stabilities of the equilibrium points can be asserted (see Tables 1 and 2), from which we can conclude that for *k* = 0.1, the equilibrium points *A*_1_, *A*_2_, *A*_3_, *A*_4_, and *A*_5_ are all unstable saddle points and for *k* = 0.5, *A*_1_ and *A*_3_ are stable center points while *A*_2_ is an unstable saddle point. It is known that equilibrium points have complicated distributions, which can result in the multistability of a chaotic system [45].

## 4. Coexistence of Multiple Firing Patterns

As it is well known, a chaotic system is sensitive to the initial value. In terms of the neuron system, the change of the initial value can make neurons demonstrate different electrical activities. To verify this result, system parameters are selected as *a* = 1.0, *b* = 3.0, *c* = 1.0, *d* = 5.0, *r* = 0.006, *S* = 4.0, *α* = 4.0, and *β* = 5.0. Coupling coefficients *k* = 0.1 and *k* = 0.5 are taken as two examples.

**Figure 9:**
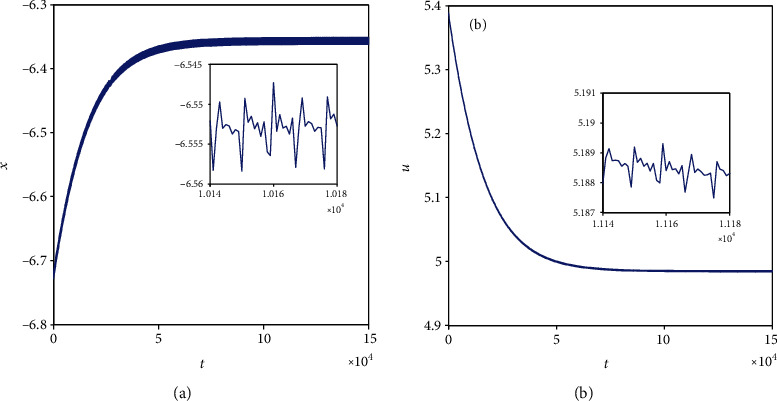
Firing patterns of membrane potentials in model (2) with *k* = 0.1, *I*_ext_ = 1.4, and initial value (−2, 0, 0, 2, 0, 0, 0).

**Figure 10:**
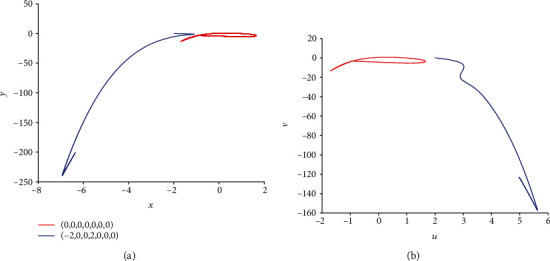
Coexistence of various firing patterns dependent on initial values in model (2) with *k* = 0.1 and *I*_ext_ = 1.4. The red phase portrait is for initial value (0, 0, 0, 0, 0, 0, 0) while the blue one is for initial value (−2, 0, 0, 2, 0, 0, 0).

**Figure 11:**
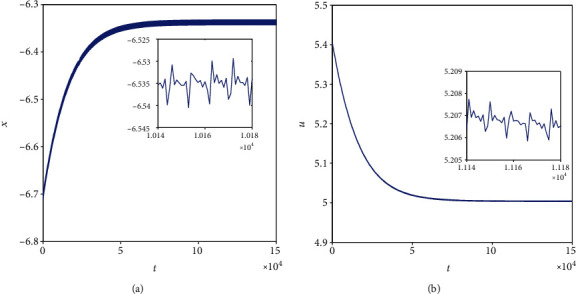
Firing pattern of membrane potential in model (2) with *k* = 0.1, *I*_ext_ = 3.3, and initial value (−2, 0, 0, 2, 0, 0, 0).

**Figure 12:**
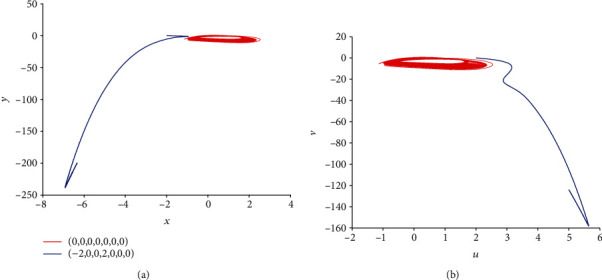
Coexistence of multiple firing patterns dependent on initial values in model (2) with *k* = 0.1 and *I*_ext_ = 3.3. The red phase portrait is for initial value (0, 0, 0, 0, 0, 0, 0) while the blue one is for (−2, 0, 0, 2, 0, 0, 0).

For the coupling coefficient *k* = 0.1 and initial value *x*_0_ = (0, 0, 0, 0, 0, 0, 0), according to Section 2.2, model (2) appears periodic firing and chaotic firing for *I*_ext_ = 1.4 and *I*_ext_ = 3.3, respectively. Namely, in model (2), various external forcing currents can lead to multiple firing patterns. In this part, the existence of multiple firing patterns of model (2) dependent on initial values is demonstrated. For this purpose, considering *I*_ext_ = 1.4, another initial value is chosen as (−2, 0, 0, 2, 0, 0, 0) and the sampled time series for membrane potential in model (2) are computed and pictured in Figure 9. Comparing Figure 1 with Figure 9, it is obvious to see that model (2) shows the period-1 firing pattern for initial value (0, 0, 0, 0, 0, 0, 0) and becomes close to being stable with small tiny vibration around equilibrium points for initial value (−2, 0, 0, 2, 0, 0, 0). The phase portrait in Figure 10 verifies the coexistence of two kinds of firing patterns. Take *I*_ext_ = 3.3 and initial value (−2, 0, 0, 2, 0, 0, 0); the sampled time series for membrane potential in model (2) are computed and drawn in Figure 11. By analyzing Figures 5 and 11, it can be found that model (2) presents a chaotic phenomenon for initial value (0, 0, 0, 0, 0, 0, 0) and tends to be stable with small tiny vibration around equilibrium points for initial value (−2, 0, 0, 2, 0, 0, 0).  Figure 12 confirms the result of Figures 5 and 11.

**Figure 13:**
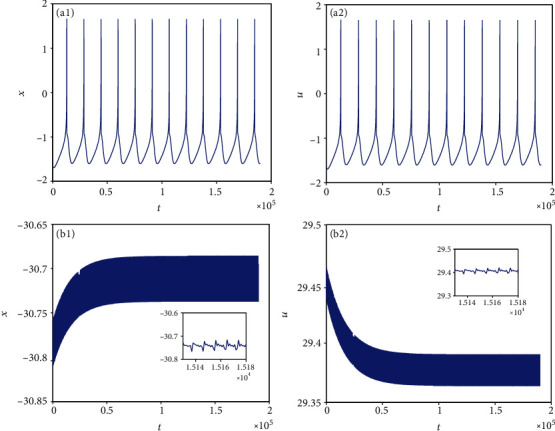
Multiple firing patterns of membrane potentials in model (2) when *k* = 0.5 and *I*_ext_ = 1.4. (a1 and a2) Time series for initial value (0, 0, 0, 0, 0, 0, 0). (b1 and b2) Time series for initial value (−2, 0, 0, 2, 0, 0, 0).

**Figure 14:**
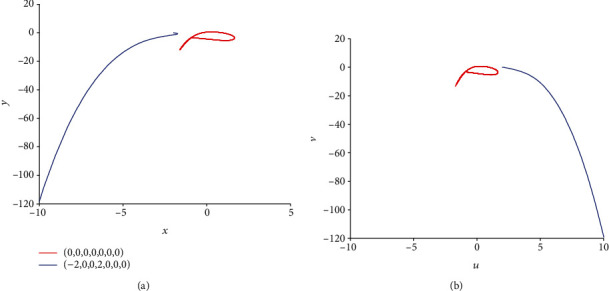
Coexistence of multiple firing patterns dependent on initial values in model (2) with *k* = 0.5 and *I*_ext_ = 1.4. The red phase portrait is for initial value (0, 0, 0, 0, 0, 0, 0) while the blue one is for (−2, 0, 0, 2, 0, 0, 0).

**Figure 15:**
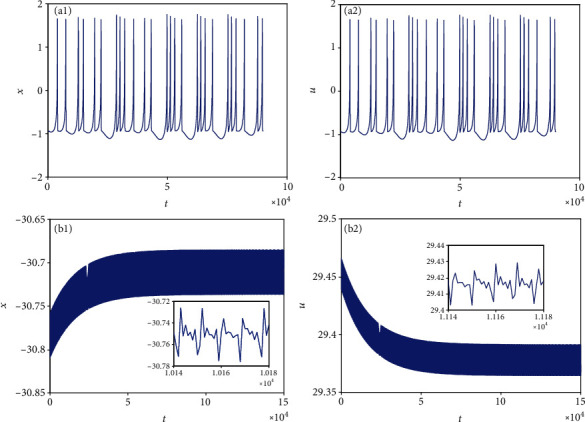
Multiple firing patterns of membrane potentials in model (2) when *k* = 0.5 and *I*_ext_ = 3.3. (a1 and a2) Time series for initial value (0, 0, 0, 0, 0, 0, 0). (b1 and b2) Time series for initial value (−2, 0, 0, 2, 0, 0, 0).

**Figure 16:**
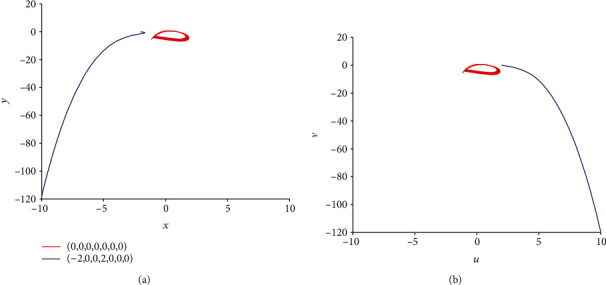
Coexistence of multiple firing patterns dependent on initial values for *k* = 0.5 and *I*_ext_ = 3.3. The red phase portrait is for initial value (0, 0, 0, 0, 0, 0, 0) while the blue one is for (−2, 0, 0, 2, 0, 0, 0).

Equally important, when *k* = 0.5, *I*_ext_ = 1.4, and initial values are selected as (0, 0, 0, 0, 0, 0, 0) and (−2, 0, 0, 2, 0, 0, 0), corresponding time series of membrane potential in model (2) are computed and pictured in Figure 13, which means that model (2) can illustrate different firing patterns for various initial values. That is to say, the coexistence of multiple firing patterns can also be disclosed, which can be tested in Figure 14. When *k* = 0.5, *I*_ext_ = 3.3, initial values are also selected as (0, 0, 0, 0, 0, 0, 0) and (−2, 0, 0, 2, 0, 0, 0), sampled time series for membrane potential in model (2) are counted and drawn in Figure 15, which shows the chaotic characteristic for initial value (0, 0, 0, 0, 0, 0, 0), while appears to be stable with small tiny vibration around equilibrium points for initial value (−2, 0, 0, 2, 0, 0, 0). It suggests that model (2) also can exhibit different firing patterns dependent on the initial values. Phase portraits in Figure 16 verify this result.

The above results indicate that when coupling coefficients are taken as *k* = 0.1 and *k* = 0.5, whether the firing pattern is periodic or chaotic, it can be changed into an approximately stable state by selecting the initial value. Namely, the coexistence of multiple firing patterns dependent on the initial values can be uncovered, while in the coupled HR model with a smooth memristor, it is hard to detect this coexistence of firing patterns.

## 5. Multiscale Effect of the Coupled HR Neuron Model by a Nonsmooth Memristor

Traditional approaches to establish a model often focus on one scale, but multiscale phenomena are part of our daily life whether we explicitly recognize it or not. For instance, as a result of the multiscale dynamics of the solar system, our time can be organized in days, months, and years. Our society is in a hierarchical structure from towns to states, countries, and continents. That is to say, it is not an easy task to think of a situation which is not involved in any multiscale characteristics. Therefore, the multiscale model is crucial in precise modeling and can provide support for the dynamic analysis of systems. For example, considering the effects of different drugs on cardiac electrophysiological activity, the drug-induced multiscale model was addressed and the mechanism of drug-induced changes in cardiac behavior was studied [46]. A multiscale model linking the cell level and the subcellular level was proposed [47], which illustrated the prediction of cancer cell migration. In terms of the inertia and response time of the hydraulic servo system, multitime scales modeling of the hydroturbine governing system was put up and the effects of time scales on the dynamical behavior of the system were analyzed [48]. Existing results suggest that the dynamics of the system are often affected by different scales and have a multiscale effect.

Encouraged by the above results, the multiscale effect of the coupled HR neuron model with a nonsmooth memristor is to be investigated. As a matter of fact, in a neuron system, electromagnetic induction current is often caused by the difference of membrane potential between two neurons. Therefore, there are different time scales in model (2). Suppose the original time scale is *t*, the fast time scale is *T*_1_, and the slow time scale is *T*_2_. Variables *x*, *y*, *z*, *u*, *v*, and *w* are related to *T*_1_, and *φ* is associated with *T*_2_. Let *T*_1_ = *t* and *T*_2_ = *εt*, and then model (2) can be rescaled as

(8) $$\begin{array}{c} \left\{\begin{array}{c} \dot{x}=y-{ax}^{3}+{bx}^{2}-z+I_{\mathrm{ext}}+kW\left(\varphi \right)\left(x-u\right),\\ \dot{y}=c-{dx}^{2}-y,\\ \dot{z}=r\left[S\left(x+1.6\right)-z\right],\\ \dot{u}=v-{au}^{3}+{bu}^{2}-w+I_{\mathrm{ext}}-kW\left(\varphi \right)\left(x-u\right),\\ \dot{v}=c-{du}^{2}-v,\\ \dot{w}=r\left[S\left(u+1.6\right)-w\right],\\ \dot{\varphi }=\varepsilon \left(\left(x-u\right)-\varphi \right), \end{array}\right. \end{array}$$

where *ε* is a small positive number on interval (0, 1), which rescales neuron model (2) into a fast subsystem and a slow subsystem under the effect of *ε*.

**Figure 17:**
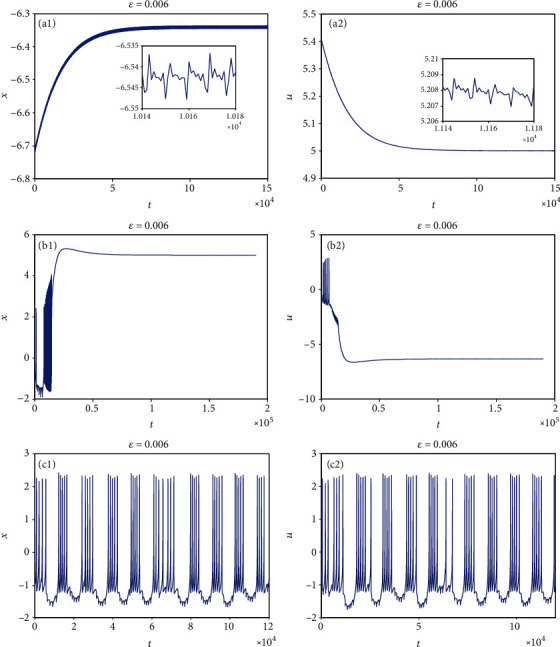
Multiple firing patterns of model (8) with small-scale *ε* changing when *k* = 0.1 and *I*_ext_ = 3.0.

**Figure 18:**
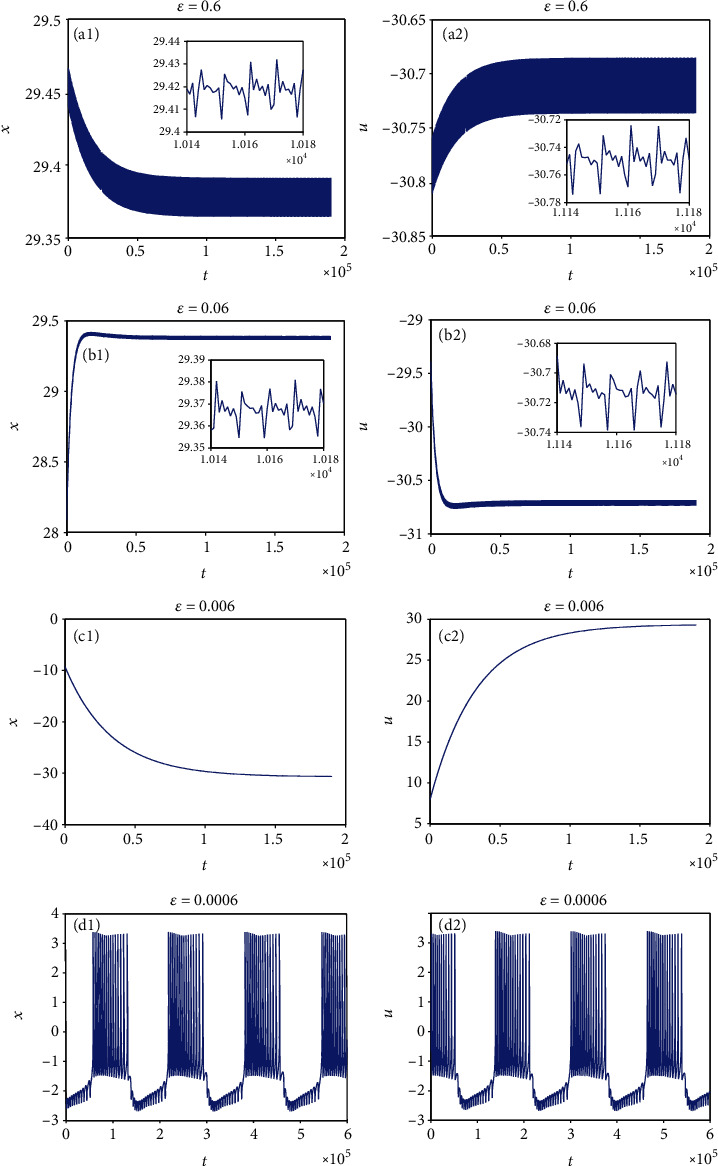
Multiple firing patterns of model (8) with small-scale *ε* changing when *k* = 0.5 and *I*_ext_ = 3.0.

System parameters are also chosen as *a* = 1.0, *b* = 3.0, *c* = 1.0, *d* = 5.0, *r* = 0.006, *S* = 4.0, *α* = 4.0, and *β* = 5.0, and the external forcing current is taken as *I*_ext_ = 3.0. When the initial value is considered *x*_0_ = (0, 0, 0, 0, 0, 0, 0), *ε* has little effect on the firing pattern of model (8). Therefore, in the following discussions, the nonzero initial value is considered, e.g., *x*_0_ = (1, 0, 0, 0, 0, 0, 0). Two cases are studied with coupling coefficients *k* = 0.1 and *k* = 0.5. The effect of small-scale *ε* on the firing pattern of model (8) is to be studied. For this purpose, *ε* is selected as different values. Sampled time series for membrane potential in model (8) are attained and given in Figures 17 and 18. From Figure 17, it can be known that for *k* = 0.1, when *ε* = 0.6, the firing patterns of membrane potentials in model (8) gradually converge to a state vibrating around a point with very low amplitude, which can be called a quasisteady state; when *ε* = 0.06, the firing patterns of membrane potentials in model (8) tend to be stable; when *ε* reduces to 0.006, the firing patterns of membrane potentials in model (8) appear a new chaotic phenomenon, in which there appears low-amplitude oscillation in the resting state between two adjacent bursting firings. It means that low-amplitude oscillation and high-amplitude oscillation can take on alternatively. From Figure 18, it is obvious to see that for *k* = 0.5, with the change of *ε*, model (8) also presents various firing patterns, which is similar to that for *k* = 0.1. Figures 17 and 18 indicate that different time scales have much effect on the firing pattern of model (8) with the nonzero initial value. Namely, model (8) has an obvious multiscale effect for the nonzero initial value. It is worth noting that compared with model (8), the firing pattern of the coupled HR model with a smooth memristor has little multiscale effect whether the initial value is zero or nonzero. This result may contribute to the optimization analysis and control of the neuron system.

## 6. Conclusions

In this paper, considering that the membrane potential difference between different neurons may be responsible for electromagnetic induction current, a neuron model coupled with a nonsmooth memristor is introduced and the multiple firing patterns are explored.

Firstly, multiple firing patterns induced by different external forcing currents are discussed. It is found that with the change of the external forcing current, the referred neuron model experiences a process of period-1, period-2, period-3, period-4, and chaos, namely, adding-period bifurcation to chaos. Secondly, the diversity of the number and stability of equilibrium points caused by the coupling coefficient is studied and it is disclosed that different coupling coefficients can make the mentioned model be provided with different numbers of equilibrium points as well as various stable states. Thirdly, the coexistence of multiple firing patterns depending on the initial value is revealed, which suggests that the dynamics of the addressed neuron model is sensitive to the initial value. Fourthly, the multiscale effect of the mentioned neuron model is uncovered and results suggest that a small scale has much effect on the firing pattern of the referred neuron model.

As has been noted, the electrical activities in coupled Hindmarsh-Rose neurons with a nonsmooth memristor display diversity. The firing pattern of it is relevant to many factors. By regulating certain factors, some required electrical activities can be achieved, which helps uncover the dynamics of neurons and then provides a basis for controlling the firing rhythm of the nervous system.

Additionally, the results in this paper can be generalized to the cases of three neurons or even more neurons, because in the real nervous system, more than two neurons can be regarded as the coupling between another neuron and the coupled neurons.

## Figures and Tables

**Table 1:** Equilibrium points, eigenvalues, and stabilities when *k* = 0.1.

No	(*δ*_1_, *δ*_2_) of the equilibrium points	Corresponding eigenvalues	Stability
*A*_1_	(-6.3408, 5.0009)	90.6301, -0.0059, -0.0063, -1.0000, -1.2470, -1.7594, -119.1816	Unstable saddle point
*A*_2_	(-1.1104, -0.439)	9.6340, 0.0839, 0.0271, -0.0050, -1.0000, -2.3443, -8.9102	Unstable saddle point
*A*_3_	(-0.7882, -0.7882)	1.1140, 0.1428, 0.0147, -0.0015, -1.0000, -4.7115, -7.7565	Unstable saddle point
*A*_4_	(-0.439, -1.1104)	9.6340, 0.0839, 0.0271, -0.0050, -1.0000, -2.3443, -8.9102	Unstable saddle point
*A*_5_	(5.0009, -6.3408)	90.6301, -0.0059, -0.0063, -1.0000, -1.2470, -1.7594, -119.1816	Unstable saddle point

**Table 2:** Equilibrium points, eigenvalues, and stabilities when *k* = 0.5.

No	(*δ*_1_, *δ*_2_) of the equilibrium points	Corresponding eigenvalues	Stability
*A*_1_	(-30.7112, 29.3776)	0, 0, -0.5, -1.0, -1.0, -1717.7, -2804.1	Stable center point
*A*_2_	(-0.7882, -0.7882)	1.1140, 0.1428, 0.0147, -1.0, -0.0015, -7.7565, -4.7115	Unstable saddle point
*A*_3_	(29.3776, -30.7112)	0, 0, -0.5, -1.0, -1.0, -1717.7, -2804.1	Stable center point

## Data Availability

The data supporting the results can be found in our paper.
